# Conversion coefficients for determination of dispersed photon dose during radiotherapy: NRUrad input code for MCNP

**DOI:** 10.1371/journal.pone.0174836

**Published:** 2017-03-31

**Authors:** Mehrdad Shahmohammadi Beni, C. Y. P. Ng, D. Krstic, D. Nikezic, K. N. Yu

**Affiliations:** 1 Department of Physics and Materials Science, City University of Hong Kong, Tat Chee Avenue, Kowloon Tong, Hong Kong; 2 Faculty of Science, University of Kragujevac, Serbia; North Shore Long Island Jewish Health System, UNITED STATES

## Abstract

Radiotherapy is a common cancer treatment module, where a certain amount of dose will be delivered to the targeted organ. This is achieved usually by photons generated by linear accelerator units. However, radiation scattering within the patient’s body and the surrounding environment will lead to dose dispersion to healthy tissues which are not targets of the primary radiation. Determination of the dispersed dose would be important for assessing the risk and biological consequences in different organs or tissues. In the present work, the concept of conversion coefficient (*F*) of the dispersed dose was developed, in which *F* = (*D*_*d*_/*D*_*t*_), where *D*_*d*_ was the dispersed dose in a non-targeted tissue and *D*_*t*_ is the absorbed dose in the targeted tissue. To quantify *D*_*d*_ and *D*_*t*_, a comprehensive model was developed using the Monte Carlo N-Particle (MCNP) package to simulate the linear accelerator head, the human phantom, the treatment couch and the radiotherapy treatment room. The present work also demonstrated the feasibility and power of parallel computing through the use of the Message Passing Interface (MPI) version of MCNP5.

## Introduction

Linear accelerators which produce photon beams are the main tools for external radiation therapy. The goal of radiation therapy is to deliver a sufficient radiation dose to the targeted tumor volume while minimizing the dose received by non-targeted healthy tissues. However, unintended radiation doses known as out-of-field doses are inevitable during the treatment, which come from scattered radiations within the patient’s body, the walls, floor and ceiling of the treatment room, and from the head of the accelerator in which the collimators are located [1–3]. The out-of-field doses in non-targeted tissues can also be affected by their distances from the treated volume.

Although unintended radiation doses are comparatively low and are neglected during most treatment planning, these have health consequences such as induction of secondary cancers [4–6]. Several international committees have advocated the need to address this issue [7–9]. Some previous studies already examined the radiation doses delivered far away from the treatment field in the patients [1, 10–12]. For example, Francois et al. [1] calculated organ doses according to their locations relative to the primary photon beam. Measurements with thermoluminescent dosimeters (TLDs) were made in an anthropomorphic water phantom to determine the dose delivered outside the beam at 10 to 50 cm away from the field edge. The authors parameterized the dose distributions for different beam energies as functions of distance from the edge, depth, field size and shape. Similarly, based on TLD measurements on the patient’s eyelids, thyroid, breast and regions of the ovary or testes, Maarouf et al. [11] examined radiation exposures of organs at risk and assessed the corresponding late effects such as secondary tumors and hereditary disorders after stereotactic radiosurgery using either a Linac or a gamma knife.

In the present work, we introduced the concept of conversion coefficient (*F*) to characterize the dose dispersion to a non-targeted tissue during a radiation therapy treatment, which was defined by *F* = (*D*_*d*_/*D*_*t*_), where *D*_*d*_ was the dispersed dose in the non-targeted tissue and *D*_*t*_ was the absorbed dose in the targeted tissue. The concept stemmed from our previous work on the determination of the conversion coefficient between the dose absorbed in an irradiated cell layer and the dose recorded by an external dosimeter [13]. For illustration purposes, five targeted tissues, namely, testes, colon, liver, left lung and brain, were studied.

Most previous works focused on modeling the dose distributions with only the beam-line-components, such as the target, primary collimator, jaws and flattening filter. The presence of the patient, which was the most significant source of scattered radiation was neglected [14–19]. On the other hand, some previous works focused on detailed simulation of the human phantom with the isocenter of a Linac, but over-simplified details in the treatment room such as the maze, primary and secondary shielding (e.g., refs. [20, 21]). Many of these results were also obtained for single or non-generic irradiation scenarios, e.g. for a specific targeted organ, which might not be readily applicable to the involved professionals. As such, the two major objectives of the present study were (1) introduction of the concept of conversion coefficients for determining dispersed doses to tissues outside the targeted volume, and (2) development of a computer code which enabled realistic simulations of the radiotherapy treatment, with consideration of an adult male human phantom adopted from the Oak Ridge National Laboratory (ORNL) [22], a Varian linear accelerator, the patient’s treatment couch and details of the treatment room.

The Monte Carlo (MC) technique has been widely employed to simulate the primary radiation treatment fields for various models of medical linear accelerators such as those from Varian, Siemens and Elekta [23, 24]. In the current study, a detailed MC model was developed to simulate the scenario where a linear accelerator (Varian Clinac 2300 C/D) was placed inside a typical treatment room with primary shielding and secondary shielding, and with a maze and an adjacent control room. A detailed male human phantom was employed to study the dose distribution among tissues, including the skeleton, skin, brain, spine, esophagus, heart, left and right lungs, within and outside the targeted volume.

## MCNP5-MPI code

The Monte Carlo N-Particle (MCNP) package has been widely used to model the radiation interaction with matter and its transport. Some of the well-known versions of this package are MCNP-4B, MCNP-4C, MCNP5 and MCNP/X. The present input code was compatible with all versions of MCNP. The current computations regarding the human phantom irradiation in a realistic radiotherapy situation were carried out using the Message Passing Interface (MPI) version of the MCNP5 program. The MPI version of the MC code enabled us to perform parallel computing on the multi-core Central Processing Units (CPUs) that enhanced the performance and the speed of the computations. Two techniques were used in the present code to minimize the relative error of the MC calculations, namely, (1) to employ a larger number of histories for each cases, and (2) to use variance reduction method by the use of increased bremsstrahlung photon multiplier number (BNUM = 40) in PHYS:E card [25, 26]. These two methods would significantly lengthen the computation time for simulation of the realistic radiotherapy scenario. Therefore, the feasibility of simulating the realistic treatment conditions using the MPI version of MCNP5 code was demonstrated, which provided a solution to provide reliable results within the shortest time.

## Modeled components

**Fig 1:**
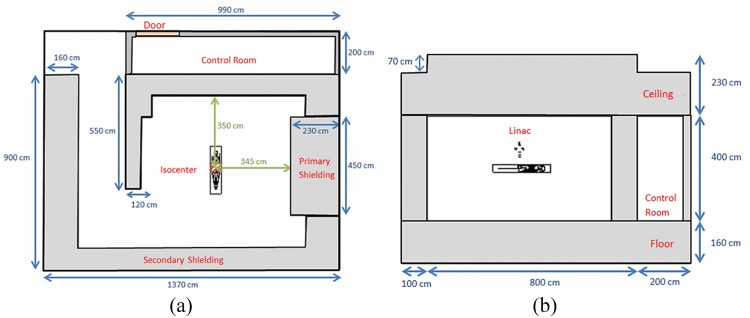
Cross-sectional views parallel to (a) y-axis and (b) x-axis of the modeled treatment room with labeled components.

The linear accelerator (Linac) model used in the present work was the commercial model of Clinac 2300 C/D from Varian Medical Systems. The developed input code consisted of a detailed Clinac 2300 C/D head with all the major components such as the primary collimators, scattering foils and the spacers. Moreover, the Linac was assumed to be operating with a voltage of 6 MV placed inside a treatment room. In addition, the patient (ORNL adult male phantom) was placed on the treatment couch below the Linac system. The room setup and the dimensions are shown schematically in Fig 1(a) and 1(b). The linear accelerator beam was directed towards the patient’s body on the treatment couch.

**Fig 2:**
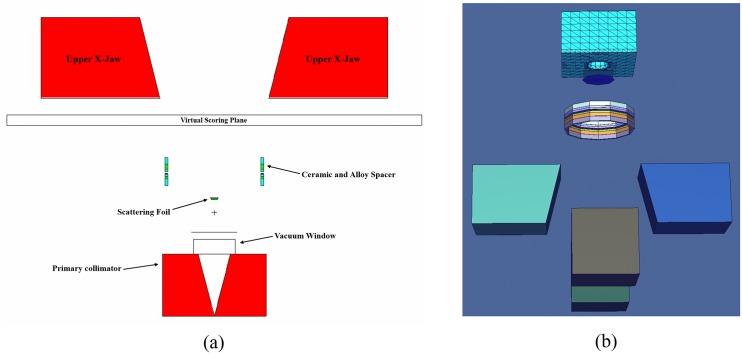
Varian Clinac 2300 C/D accelerator head view from (a) x-y plane and (b) 3-dimensional snapshots. (Note: only a few major parts visible from the x-y plane were labeled).

Fig 2(a) shows the major components of the Linac head, namely, the primary collimator, vacuum window, scattering foil, ceramic and alloy spacing and the upper X-jaws. In addition, Fig 2(b) provides the three-dimensional view of the Linac head, which helps visualization of the modeled components. The treatment room had dimensions 13.7 × 9 × 4 m^3^ and was shielded with ordinary concrete with density = 2.35 g/cm^3^. The maze was located to the left of the room with height and width of 2.2 and 2 m, respectively. The isocenter of the Varian 2300 C/D Linac was located at the center of the treatment room where the patient phantom was placed under the linear accelerator with a source-to-isocenter distance of 100 cm. Moreover, in order to emulate the realistic radiotherapy scenarios, a control room with a door next to the treatment room was developed with a volume of 9.9 × 2 × 4 m^3^. The adult male phantom model used in the present work was adopted from ref. [27]. The phantom geometry and material properties assigned to different parts of the patient’s body were kept unchanged. The surrounding medium filling the treatment room was modeled as air at room temperature and pressure (air = 0.00129 g/cm^3^).

## Computation scheme

**Fig 3:**
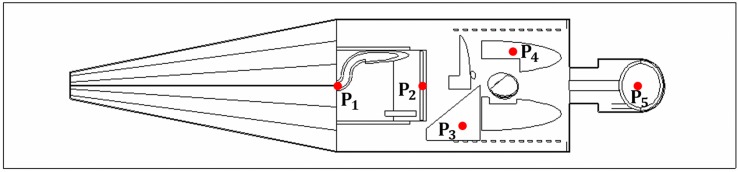
The five irradiation positions (*P*_1_, *P*_2_, *P*_3_, *P*_4_ and *P*_5_) chosen as targets in the present study. The red dots represent the positions of irradiation.

Five different targets, namely, testes (*P*_1_), colon (*P*_2_), liver (*P*_3_), left lung (*P*_4_) and brain (*P*_5_) were chosen as the targets, and their positions are schematically shown in Fig 3.

**Fig 4:**
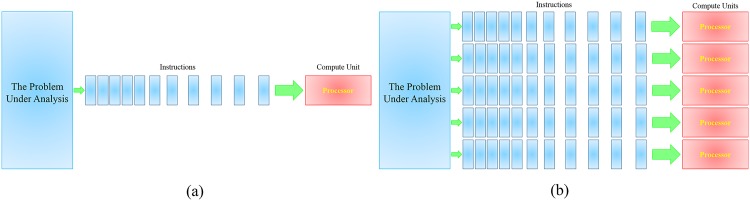
Simplified schematic diagram showing the concept of (a) serial (conventional) computing and (b) parallel computing using multi-processor compute units.

The present computations were performed on a supercomputer which consisted of dual Intel Xeon E5-2630 v3 2.40 GHz CPUs. The system consisted of 16 physical CPU cores hyper-threaded to 32 cores for the current study. The MCNP5-MPI code was launched using the *mpiexec* command which was supplied by the MPICH2 package for the purpose of parallel codes execution [28]. The schematic diagram shown in Fig 4 demonstrates the main difference between the serial and parallel computation. The serial computation (Fig 4a) breaks the problem into instructions that will be executed on a single processor. This is desirable for programs that are having relatively simplified physics and geometry therefore the computation time will be maintained within the feasible limit. On the other hand, the parallel computation (Fig 4b) is useful and sometimes even vital for computer programs that are having a large number of components. The current input code consists of a large number of details that will lengthen the computation time so the use of parallel computation is highly recommended.

## Absorbed dose (*D*_*t*_) in targeted tissues and dispersed dose (*D*_*d*_) in non-targeted tissues

The dose delivered to the targeted and non-target tissues from the photon beam was calculated using the track length estimate of energy tally. Eq (1) was used to determine the total energy deposited (*H*_*total*_) in the domain representing the tissue (targeted or non-targeted tissue) under analysis:

$$\begin{array}{c} H_{total}=\frac{\rho_{\alpha }}{m}\int dE\int dt\int dV\int d\Omega \sigma_{t}\left(E\right)H\left(E\right)\Psi \left(r,\Omega ,E,t\right) \end{array}$$ (1)

where *σ*_*t*_ was the total energy-dependent photo-atomic microscopic cross-section for each specific nucleus present in the tissue under analysis. The ENDF/B.VI release 8 photo-atomic data were used in the present model. The scored dose was computed using tally F6:P with the unit of MeV/g, which could be converted into Gy.

**Fig 5:**
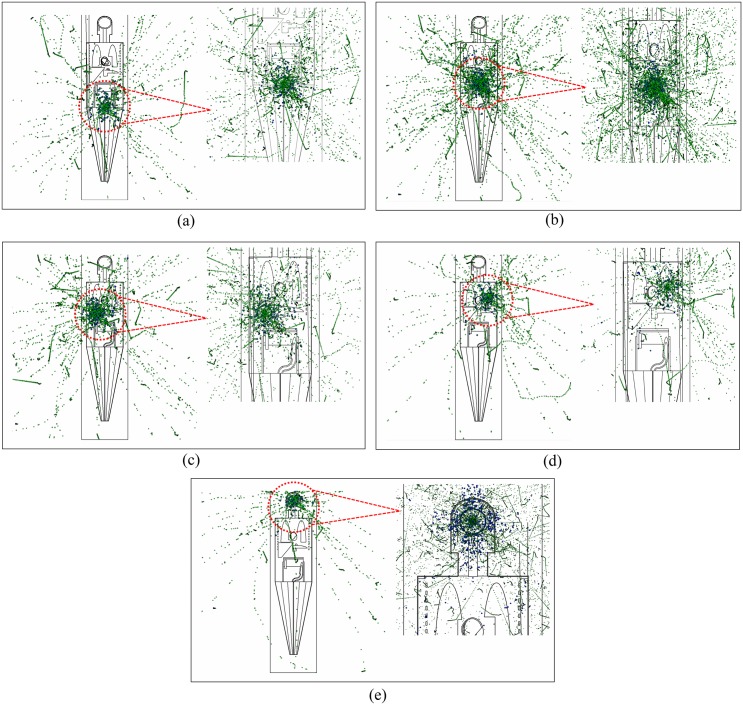
Dispersion of primary photons (shown in blue) and secondary electrons (shown in green) during the radiotherapy treatment at different irradiation positions: (a) testes (*P*_1_), (b) colon (*P*_2_), (c) liver (*P*_3_), (d) left lung (*P*_4_) and (e) brain (*P*_5_). The snapshots were generated using the Vised visualizer.

The output results for the conversion coefficient *F* given by the MC program were normalized by the number of primary particles so *F* would be independent of the particle fluence in the tissue under analysis. The dispersions of particles within the human phantom were graphically depicted in Fig 5(a)–5(e) for the five irradiation positions considered in this work.

The snapshots were generated using the Vised visualizer (bundled with MCNP) at different irradiation positions as shown in Fig 3. The photon and electron tracks were separately shown as blue and green dots, respectively. The enlarged sections provided better views of the targets and their surrounding tissues during irradiation. It is remarked there that photon interactions with matter involve atomic electrons, so each blue dot (photon hit with interaction) is associated with at least one green dot (electron hit with interaction).

## Results and discussion

One of the important components in the present input code was the linear accelerator head which collimated the photon beam. Therefore, the Linac head was benchmarked with experimental data in the literature. Bencheikh et al. (2016) studied the percent depth dose (PDD) for the two widely used Varian Clinac 2100 and 2300 accelerators [29]. The PDD was the ratio between the dose *D*_*i*_ measured at a depth (i) and the maximum dose *D*_*max*_ on the beam central axis with a specified field size, i.e.,

$$\begin{array}{c} PDD=\frac{D_{i}}{D_{max}} \end{array}$$ (2)

**Fig 6:**
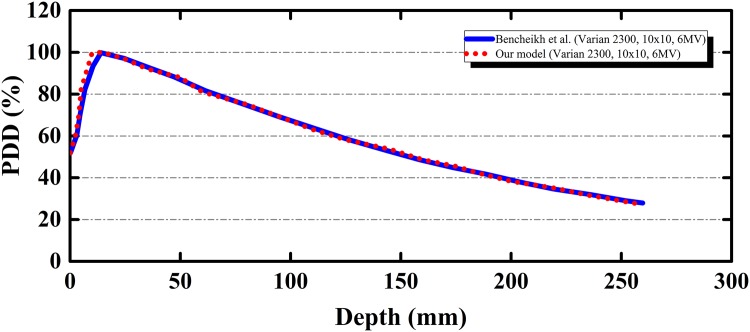
Benchmarking of the present model against the experimental data from Varian Clinac 2300 linear accelerator model through percent dose depth (PDD). (Solid line: experimental data; dotted line: our model).

For benchmarking purposes, the accelerator set at 6 MV was used to irradiate a water phantom with a measurement depth of 30 cm, a source-to-surface distance (SSD) of 100 cm, and a field size of 10 × 10 cm^2^. The benchmarking results are shown in Fig 6 and agreement between the modeled and experimental results was apparent.

**Fig 7:**
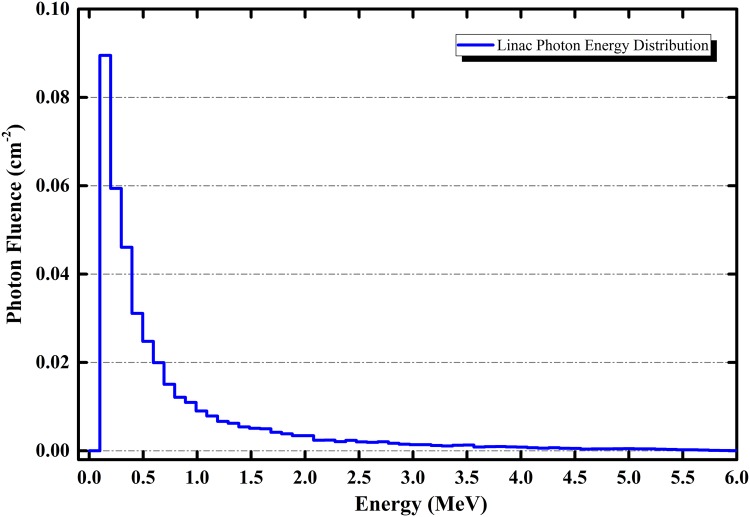
Photon energy distribution exiting from the linear accelerator operating at 6 MV after beam filtration and collimation.

The distribution of photon energy exiting from the accelerator operating at 6 MV is shown in Fig 7. These results were scored over the outer boundary of the virtual scoring plane shown in Fig 2(a). From the photon spectrum shown in Fig 7, the average energy of the photons striking the targeted tissues in the human phantom was ∼0.5 MeV.

**Fig 8:**
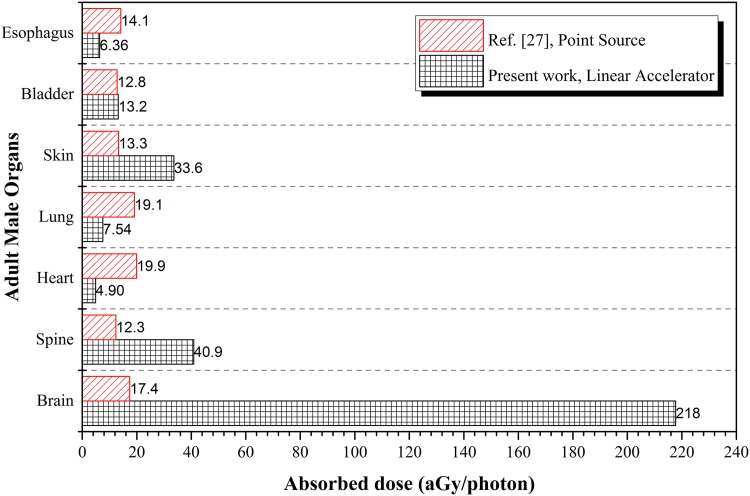
Comparison between the absorbed doses in major tissues of the adult male phantom during head irradiation obtained using a ^137^*Cs* point source [27] and a 6 MV linear accelerator beam in the present work. The results from irradiation at the brain (*P*_5_) in the present work were used for comparison with the results in ref. [27].

This average energy was also close to the photon energy (E = 661.6 keV) used by Krstic and Nikezic from a monoenergetic ^137^*Cs* point source [27]. The similar photon energies allowed comparisons between the resulting doses in the major tissues, using the results from irradiation at the brain (*P*_5_) in the present work for comparison and noting that the results from ref. [27] for irradiation of the face. The comparisons between the absorbed doses in different tissues are shown in Fig 8. Apparently, the linear accelerator delivered more energy into the tissues located near the head and neck (closer to point of irradiation).

The absorbed dose in brain was 218 aGy/photon from Linac irradiation, but the value was only 17.4 aGy/photon from the ^137^*Cs* point source. The reasons behind the large difference were mainly two fold. First, the collimated beam was more effective in penetrating the skull to deliver a larger amount of energy into the targeted tissues. Second, the lower-energy photons (E ≤ 0.1 MeV) in the Linac beam could lose a larger fraction of their energies through the photoelectric effect, in contast to the higher-energy photons (E > 0.1 MeV) which would lose a smaller fraction of their energies through Compton scattering. On the other hand, the spine was located close to the phantom head so the dose delivered by the Linac was expected to be larger than that delivered by the point source, which was indeed observed, viz., 40.9 and 12.3 aGy/photon from the Linac and point sources, respectively. In contrast, for tissues located further away from the irradiation position, the point source would be expected to deliver larger doses due to absence of beam collimation and more scattering as a result of Compton scattering. This was confirmed by the absorbed dose in esophagus, lung and heart: 6.36, 7.54 and 4.90 aGy/photon, respectively, from Linac, and 14.1, 19.1 and 19.9 aGy/photon, respectively, from the point source. The present results also highlighted that use of over-simplified sources for modeling could potentially lead to inaccurate results. The absorbed doses in the targeted tissues for various irradiation positions *P*_1_ to *P*_5_ are shown in Table 1, which can be employed to determine the conversion coefficients *F* for the dispersed dose in the non-targeted tissues.

**Table 1:** Absorbed doses in targeted tissues for different irradiation positions *P*_1_ to *P*_5_ using the 6 MV linear accelerator beam.

Irradiation position	*P*_1_	*P*_2_	*P*_3_	*P*_4_	*P*_5_
Targeted tissue	Testes	Colon	Liver	Left Lung	Brain
Absorbed dose (aGy/photon)	361	195	250	243	217

**Fig 9:**
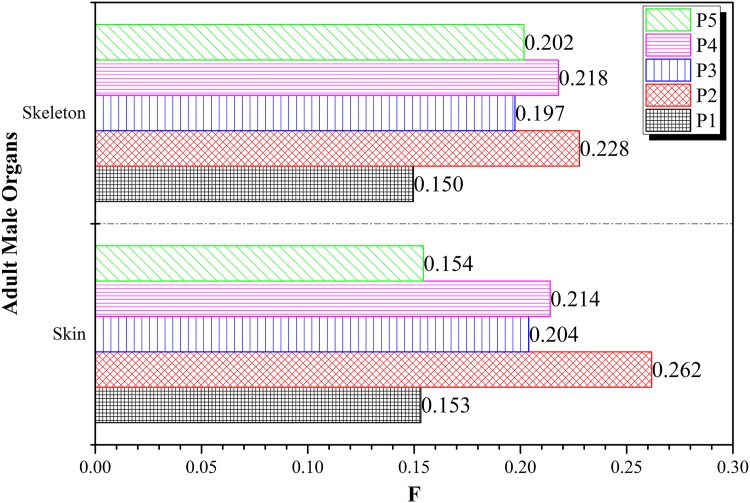
Variations in *F* for the skin and skeleton obtained at different irradiation positions *P*_1_ to *P*_5_ using the 6 MV linear accelerator beam.

Fig 9 shows the variations in *F* for skin and skeleton obtained at different irradiation positions *P*_1_ to *P*_5_, which were not significant. Therefore, the doses delivered to the skin and skeleton would be largely independent of the irradiation position when collimated beams from the Linac were used during the treatment. The largest and smallest *F* values in the skeleton were 0.228 and 0.150 corresponding to *P*_2_ and *P*_1_, respectively; those in the skin were 0.262 and 0.153 also corresponding to *P*_2_ and *P*_1_, respectively. The insignificant variations in the *F* values for the skin and skeleton were mainly due to the pervasive nature of these tissues over the entire human body so the dependence on the irradiation position was expected to be small.

**Fig 10:**
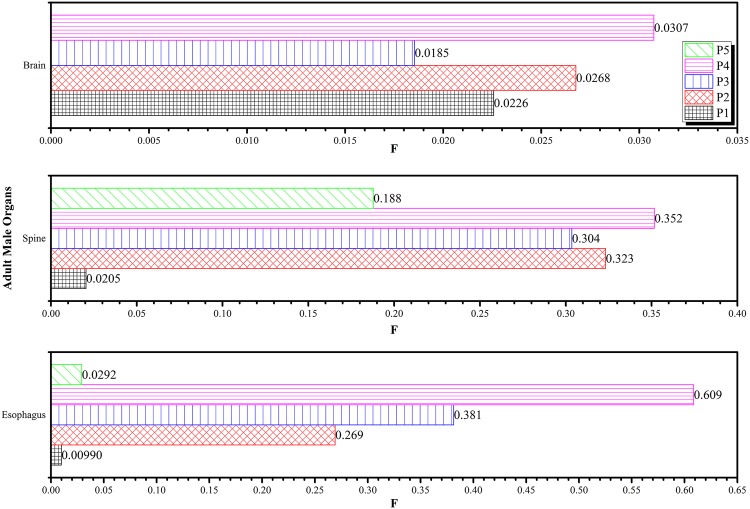
Variations in *F* for the brain, spine and esophagus obtained at different irradiation positions *P*_1_ to *P*_5_ using the 6 MV linear accelerator beam.

**Fig 11:**
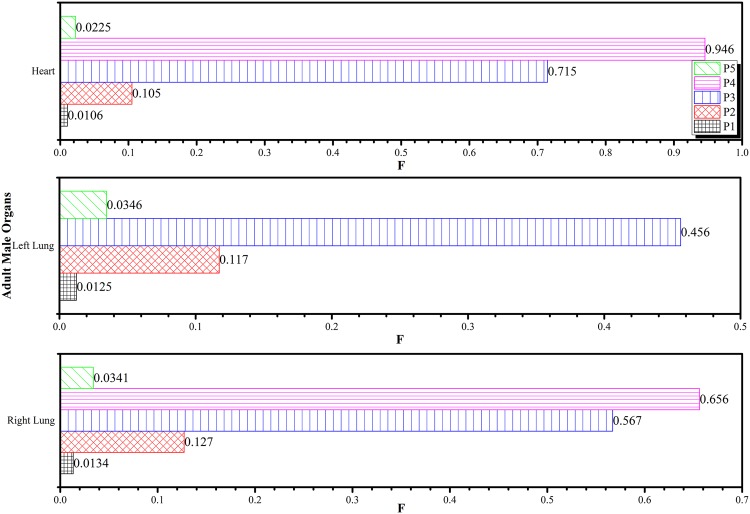
Variations in *F* for the heart, left and right lung obtained at different irradiation positions *P*_1_ to *P*_5_ using the 6 MV linear accelerator beam.

**Fig 12:**
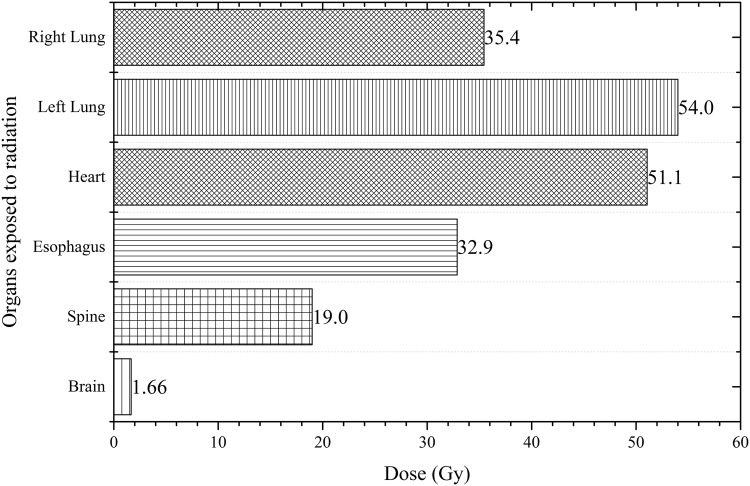
Dispersed doses delivered to non-targeted tissues in an example which determined the dispersed doses during a CHART (continuous hyper-fractionated accelerated radiotherapy) treatment of lung cancer.

Similarly, Fig 10 shows the variations in *F* for the brain, spine and esophagus while Fig 11 shows the variations in *F* for the heart, left and right lung obtained at different irradiation positions *P*_1_ to *P*_5_ using the 6 MV linear accelerator beam. By definition, the left lung and brain had *F* = 1 for irradiation positions at *P*_4_ and *P*_5_, respectively, so these were not shown in the figures. Among the tissues in Figs 10 and 11, the largest *F* values all occurred when *P*_4_ (left lung) was irradiated. Although the *F* values for tissues were expected to decrease with their distances from the irradiation position, the patterns in Figs 10 and 11 showed more stochastic variations as a result of scattering of the primary photons and secondary electrons within the human phantom. This highlighted the importance of developing an input code for determining the *F* values for chosen irradiation positions. During the real-life treatment, it would be difficult to experimentally measure the dispersed doses within the patient’s body. By using the *F* values computed in the present work, the doses delivered to non-targeted tissues could be determined. The following gave an example to illustrate the usefulness of the *F* value, which determined the dispersed doses during a CHART (continuous hyper-fractionated accelerated radiotherapy) treatment. The treatment schedule which delivered 54 Gy to the targeted region within the lung in 36 fractions, with 3 fractions per day over 12 days [30, 31] was considered. Through the conversion coefficients determined in the present work, the dispersed doses delivered to the right lung, heart, esophagus, spine and brain were obtained as shown in Fig 12.

The doses absorbed by the heart and right lung were 51.1 and 35.4 Gy, respectively, showing that the heart would receive the second highest dose upon the irradiation of left lung during the treatment. The spine is an organ which requires special attention since it requires a relatively long recovery time after treatment. The dose delivered to this organ was 19 Gy for the CHART example here. In addition, it is remarked here that the statistical uncertainties of all results presented in this work were determined to be below ∼0.4%.

## Conclusions

The present work showed the significance of dispersed doses delivered to non-targeted tissues during radiotherapy treatment of a targeted tumor. An input code was developed to determine the conversion coefficients which could be readily used to determine the dispersed doses. The present model showed that the doses delivered by the collimated beam were significantly different from those estimated using a simplified point source. The doses delivered to the skeleton and skin were largely independent of the irradiation positions. For illustration purposes, the present work determined the doses dispersed to non-targeted tissues such as the spine, esophagus, heart, left and right lung during a CHART (continuous hyper-fractionated accelerated radiotherapy) treatment of the left lung. In general, the dose dispersed to a non-targeted tissue decreased with its distance from the irradiation position (left lung). For example, the doses delivered to the heart (closer to the left lung) and right lung (more distant from the left lung) were 94.63 and 65.56%, respectively, of the total dose delivered to the left lung. Similarly, the doses delivered to the esophagus (closer) and spine (more distant) were 60.93 and 35.19%, respectively, of the total dose delivered to the left lung. Doses were delivered to non-targeted tissues due to scattering of primary photons and secondary electrons. Furthermore, the feasibility of simulating the realistic treatment conditions using parallel (MPI) computing was assessed through the MCNP5-MPI program. The success provided a solution to provide reliable results within the shortest time.
